# Dermal fibroblast-like cells reprogrammed directly from adipocytes in mouse

**DOI:** 10.1038/s41598-020-78523-8

**Published:** 2020-12-08

**Authors:** Mitsunobu Toyosaki, Koichiro Homma, Sayuri Suzuki, Naoto Muraoka, Hisayuki Hashimoto, Naoki Goshima, Masaki Ieda, Junichi Sasaki

**Affiliations:** 1 Department of Emergency and Critical Care Medicine, Keio University School of Medicine, 35 Shinanomachi, Shinjuku-ku, Tokyo 160-8582 Japan; 2 Department of Cardiology, Faculty of Medicine, Keio University School of Medicine, 35 Shinanomachi, Shinjuku-ku, Tokyo 160-8582 Japan; 3 Molecular Profiling Research Center for Drug Discovery, National Institute of Advanced Industrial Science and Technology, AIST Tokyo Waterfront Bio-IT Research Building 2-4-7 Aomi, Koto-ku, Tokyo 135-0064 Japan; 4 Department of Cardiology, Faculty of Medicine, University of Tsukuba, 1-1-1 Tennodai, Tsukuba, Ibaraki 305-8575 Japan

**Keywords:** Developmental biology, Reprogramming, Diseases, Skin diseases

## Abstract

In deep burns, early wound closure is important for healing, and skin grafting is mainly used for wound closure. However, it is difficult to achieve early wound closure in extensive total body surface area deep burns due to the lack of donor sites. Dermal fibroblasts, responsible for dermis formation, may be lost in deep burns. However, fat layers composed of adipocytes, lying underneath the dermis, are retained even in such cases. Direct reprogramming is a novel method for directly reprograming some cells into other types by introducing specific master regulators; it has exhibited appreciable success in various fields. In this study, we aimed to assess whether the transfection of master regulators (ELF4, FOXC2, FOXO1, IRF1, PRRX1, and ZEB1) could reprogram mouse adipocytes into dermal fibroblast-like cells. Our results indicated the shrinkage of fat droplets in reprogrammed mouse adipocytes and their transformation into spindle-shaped dermal fibroblasts. Reduced expression of PPAR-2, c/EBP, aP2, and leptin, the known markers of adipocytes, in RT-PCR, and enhanced expression of anti-ER-TR7, the known anti-fibroblast marker, in immunocytochemistry, were confirmed in the reprogrammed mouse adipocytes. The dermal fibroblast-like cells, reported here, may open up a new treatment mode for enabling early closure of deep burn wounds.

## Introduction

The skin has two primary layers, the outer epidermis and inner dermis. The latter is composed of cells, fibers, vessels, nerves, and glands. Although superficial burns do not involve the dermis and are healed without surgical treatments, deep burns (partial-thickness, full-thickness, and deeper burns) do involve the dermis, and many of them require surgical treatments to heal. Surgical treatments for deep burns include burn wound excisions and coverage. Early burn wound excisions are important for eliminating necrotic and potentially infected tissues^1,2^. Some randomized studies have shown early burn wound closure to decrease scar thickness^3^ and joint contractures, and shorten the hospital stay^4^. Direct wound closure, skin grafting, tissue expansion, and flap reconstruction are used for burn wound closure, and skin grafting especially plays an important role in large-total body surface area (TBSA) deep burns. However, in extensive TBSA deep burns (especially full-thickness and deeper burns), it is difficult to achieve early burn wound closure owing to the lack of donor sites.

In many full-thickness or deeper burns, fat layers composed of adipocytes, lying underneath the dermis, are safely retained. Adipocytes originate from stromal cells such as dermal cells. Production of dermal fibroblasts, responsible for the formation of dermis, using the safely maintained self-adipocytes, may be helpful in deep burn care. However, there has been no prior study using self-adipocytes, either in vivo or in vitro.

Direct reprogramming is a novel method to directly reprogram some cells into other types by introducing master regulators, and has achieved appreciable success in various fields^5–8^. For instance, cardiomyocytes have been successfully reprogrammed from fibroblasts by introducing three developmental transcription factors^5^. Although using the direct reprogramming technique in instances of burns may be challenging, successful reprogramming of adipocytes into dermal fibroblasts can alter burn treatment.

In this study, we examined whether six master regulators (ELF4, FOXC2, FOXO1, IRF1, PRRX1, and ZEB1), selected using Mogrify (https://mogrify.co.uk/), could reprogram adipocytes differentiated from OP9 cells into dermal fibroblasts.

## Results

### Cell culture of adipocytes

**Figure 1:**
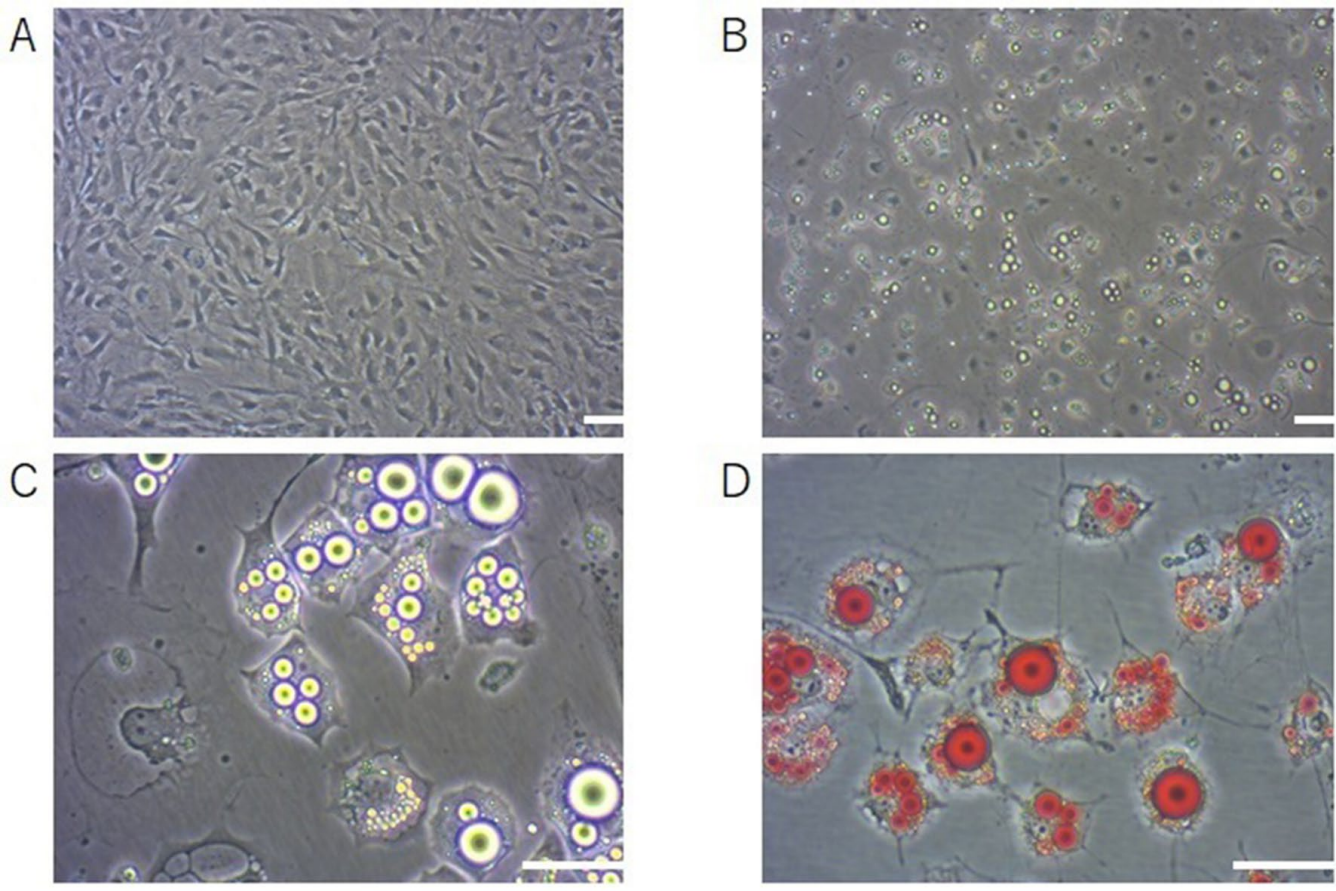
Differentiation of adipocytes from OP9 cells. (**A**–**D**) OP9 cells were cultured (**A**), and adipocytes differentiated from them (**B**,**C**) were stained with Oil Red O. Lipid droplets are highlighted (**D**). Scale bars, 100 µm (**A**,**B**) and 50 µm (**C**,**D**).

Adipocytes were successfully differentiated from OP9 cells, with differentiation rates between 70 and 80%. They were stained with Oil Red O, and formed noticeable fat droplets (Fig. 1).

### Assessment of morphological changes

The IncuCyte system was used to observe the sequential morphological changes. To distinguish transfected cells from others, GFP was used along with the six master regulators.

**Figure 2:**
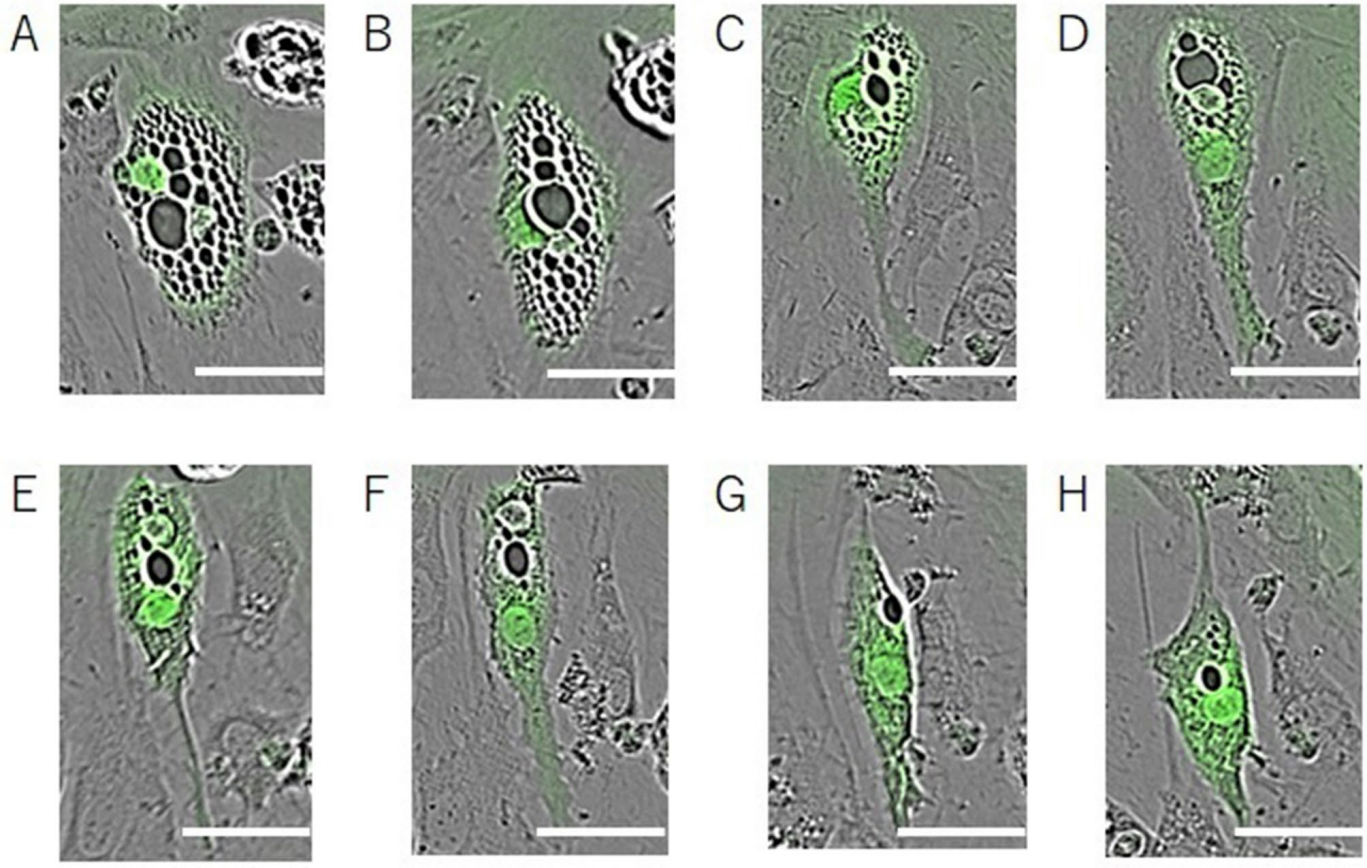
Shape change of infected cells (**A**–**H**). At 120 h after transfection, no marked change was observed (**A**). After 126 h, cells became vertically elongated (**B**). At 159 h (**C**), 168 h (**D**), 189 h (**E**), and 198 h (**F**) after transfection, the cells were further elongated vertically, and fat droplets were shrunk (**C**–**F**). After 225 h (**G**) and 237 h (**H**) of transfection, cells became spindle-shaped, just like fibroblasts, and fat droplets almost disappeared (**G**,**H**). Scale bars, 50 µm.

Sequential morphological changes in one of the transfected cells are shown in Fig. 2. In this cell, no marked change was observed until 120 h after transfection, and marked changes were noted after 120 h. First, the cell changed from a round shape to a vertically elongated shape. Second, fat droplets shrunk gradually and the cell changed into a yet longer shape. Finally, fat droplets almost disappeared and the cell changed into a spindle-shaped fibroblast (Fig. 2).

These changes were not observed in all GFP-positive cells, which could be due to dispersion of transfections. However, it is not yet clear whether all the six master regulators were successfully transfected into all the GFP-positive cells. In some cells, only GFP might have been transfected, while the six master regulators were not.

Based on the above, we confirmed that a transfected cell markedly changes into a spindle-shaped fibroblast, with shrunk fat droplets.

### Quantitative RT-PCR

**Figure 3:**
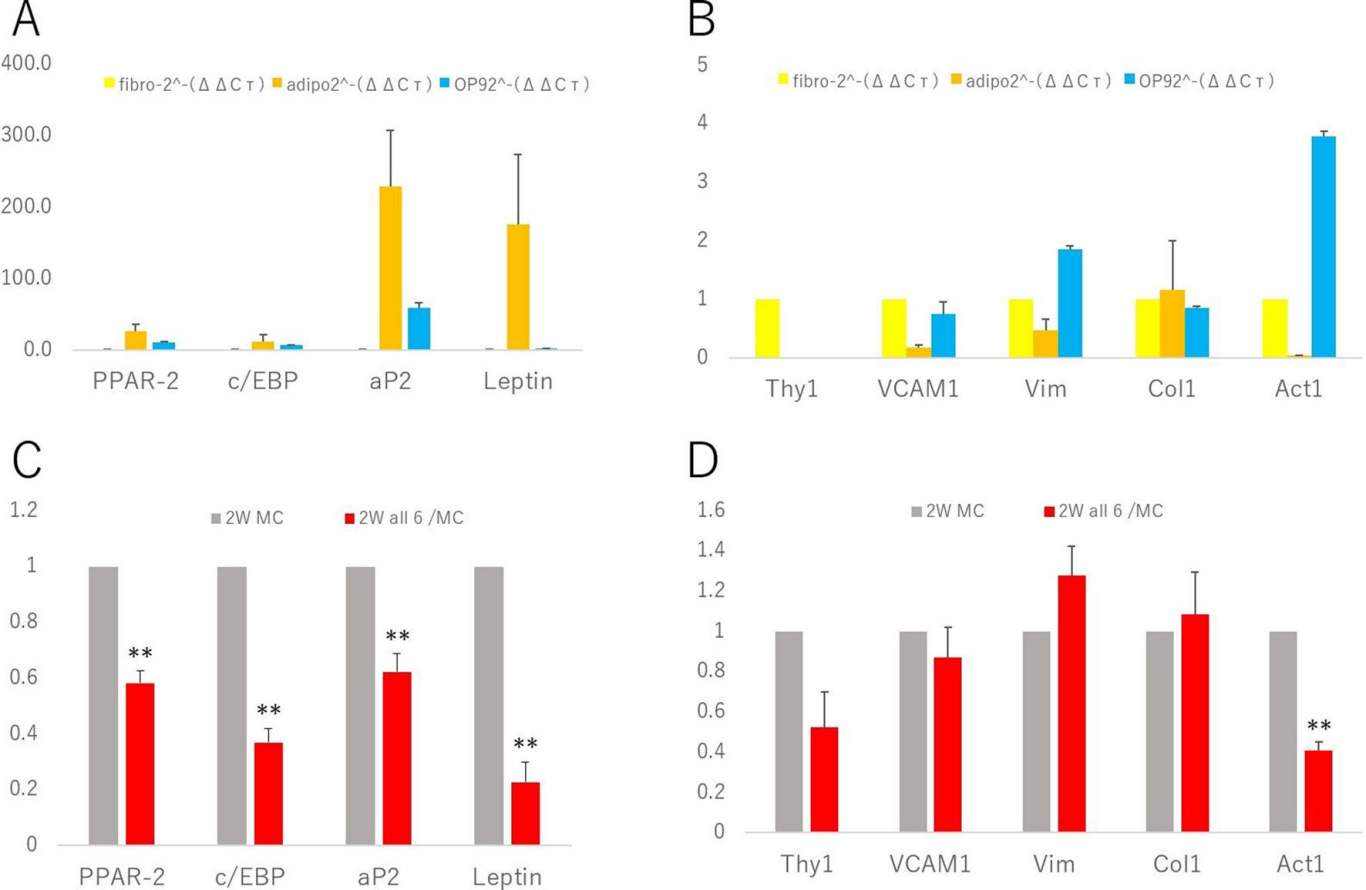
Quantitative data of gene expression. (**A**–**D**) Four candidate genes as markers of adipocytes. All of them were expressed at higher levels in adipocytes than in OP9 cells and fibroblasts (**A**). Five candidate genes as markers of fibroblasts. Thy1 expression was higher in fibroblasts than in OP9 cells and adipocytes. Col1 was expressed in any of the cells, with no distinct difference. VCAM1, Vim, and Act1 were expressed at higher levels in fibroblasts and OP9 cells than in adipocytes (**B**). The expression of four candidate genes as markers of adipocytes in negative control (MC: Medium Change) cells and transfected (all of the 6 master regulators) cells after 2 weeks of culture (**C**). The expression of five candidate genes as markers of fibroblasts in MC cells and transfected cells after a 2-week culture (**D**). (n = 8). Values shown are the means + SEM. P-values were determined by a Student’s t-test. **P < 0.01.

Four candidate genes (PPAR-2, c/EBP, aP2, and leptin) that are well-known as markers of adipocytes were confirmed to be expressed at higher levels in adipocytes than in OP9 cells and fibroblasts; positive control cells originated from mouse tails. All these genes seemed to be important markers to distinguish adipocytes from other cells (Fig. 3A).

We checked five candidate genes (Thy1, VCAM1, vimentin, collagen 1, and actin) as markers of fibroblasts. Unlike those of adipocytes, specific gene markers of fibroblasts are not well-known, and our selected candidate genes showed differential expression patterns. Thy1 expression was higher in fibroblasts than in OP9 cells and adipocytes. Although Col1 was expressed in any of the cells, with no distinct differences, VCAM1, vimentin, and actin were expressed at higher levels in fibroblasts and OP9 cells than in adipocytes. Among these five candidate genes, Thy1 seemed to be a strong marker for distinguishing fibroblasts from other cells (Fig. 3B).

The expression of candidate gene markers for adipocytes and fibroblasts was checked in transfected cells and negative control cells. Cells transfected with all the six master regulators were cultured for 2 weeks (all 6 cells). Negative control cells were also cultured for 2 weeks, and during the culture periods, only medium changes were performed (MC cells). All four candidate genes for adipocytes were expressed at higher levels in MC cells than in all 6 cells, and the differences were significant (P = 0.004, 0.00002, 0.002, and 0.0004 for PPAR2, c/EBP, ap2, and leptin, respectively) (Fig. 3C). On the other hand, the expression of five candidate genes for fibroblasts showed various patterns. Vimentin and collagen 1 were expressed at higher levels in MC cells than in transfected cells, although the differences were not very clear (P = 0.2 and 0.9 for vimentin and collagen 1, respectively), while VCAM1 and actin showed reverse results. Thy1 seemed to be a strong marker. Only differences in actin expression levels were significant (P = 0.2, 0.2, and 0.00004 for Thy1, VCAM1, and actin, respectively) (Fig. 3D).

Based on these results, all 6 cells seemed to change from adipocytes after transfection by the six master regulators; however, our results could not show a clear evidence of them being changed to fibroblasts. Thy1, one of the candidate genes, seemed to be a strong marker for fibroblasts.

Since these results were quantitative, infection rates could strongly affect the results. Therefore, increasing the transfection rates would be an important task in the future as it would enable the provision of a strong evidence for the higher expression of Thy1 in infected cells than in MC cells, with clear differences.

### Immunofluorescence

Anti-ER-TR7, as a fibroblast marker, and anti-FABP4, as an adipocyte marker, were used for immunofluorescence staining. Hoechst 33258 was used for visualizing the nuclei.

**Figure 4:**
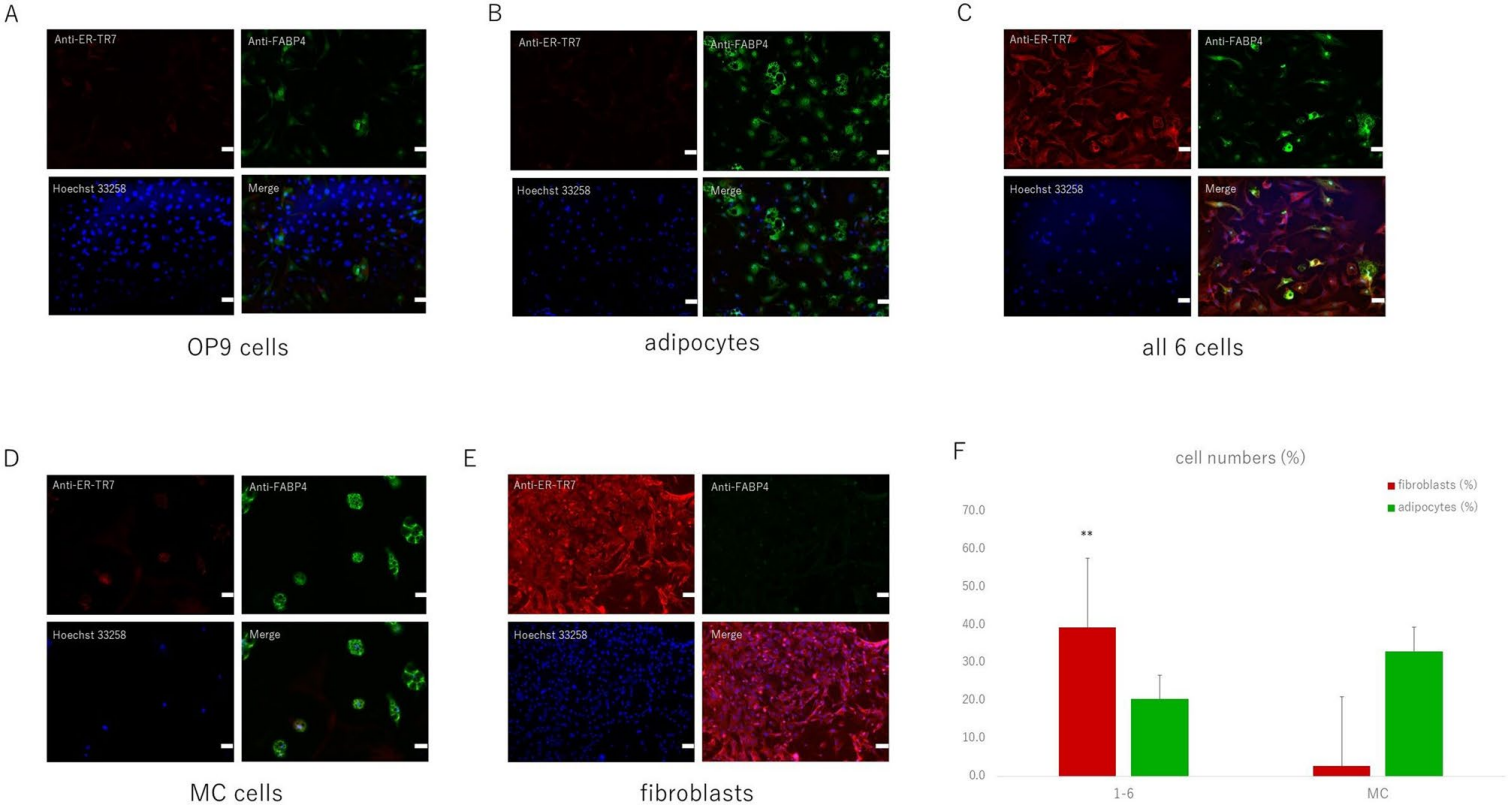
Immunofluorescence staining for anti-ER-TR7 (red) and anti-FABP4 (green). Hoechst 33258 (blue) was used for visualizing nuclei. (**A**–**E**) Combination of anti-ER-TR7 and anti-FABP4 expression in OP9 cells (**A**) and in adipocytes (**B**). Anti-ER-TR7 and anti-FABP expression was less in OP9 cells, whereas anti-FABP expression was appreciable in adipocytes. Expression of anti-ER-TR7 and anti-FABP4 in all 6 cells (**C**), MC cells (**D**), and fibroblasts (**E**). Anti- ER-TR7 expression was higher in all 6 cells and fibroblasts than in MC cells, whereas anti-FABP4 expression showed reverse results. Scale bars, 50 µm. The number of fibroblasts, stained by anti-ER-TR7, and number of adipocytes, stained by anti-FABP4, in all 6 cells and MC cells are showed as a percentage. The percentage of fibroblasts in all 6 cells was much more than that in MC cells (**F**). (n = 10) Values shown are the means + SEM. P-values were determined by a Student’s t-test. **P  < 0.01.

First, the combination of anti-ER-TR7 and anti-FABP4 expression in OP9 cells and adipocytes was checked. In OP9 cells, both anti-ER-TR7 and anti-FABP4 were expressed at low levels (Fig. 4A). On the other hand, in adipocytes differentiated from OP9 cells, anti-FABP4 expression was higher than that of anti-ER-TR7 (Fig. 4B).

Second, the expression of anti-ER-TR7 and anti-FABP4 was checked in “all 6 cells”; all 6 genes were used for infection, and “MC cells” were cultured for 2 weeks. During the culture periods, only the medium was changed routinely. In case of fibroblasts, positive control cells originating from mouse tails were cultured for 2 weeks. In all 6 cells (Fig. 4C), and fibroblasts (Fig. 4E), a higher anti-ER-TR7 expression was confirmed, while anti-FABP expression was low. In MC cells (Fig. 4D), similar to the results of adipocytes differentiated from OP9 cells, anti-FABP4 expression was substantially higher than that of anti-ER-TR7.

The number of fibroblasts stained by anti-ER-TR7 and adipocytes stained by anti-FABP4 in all 6 cells and MC cells was counted and presented as a percentage in the graph. Percentage of fibroblasts in all 6 cells was considerably more than that in MC cells (all 6 cells: 39.4%, MC cells: 2.6%), and the differences were significant (P = 0.003), whereas that of adipocytes in MC cells was more than that in all 6 cells (all 6 cells: 20.4%, MC cells: 33.0%), although the differences were not significant (P = 0.3) (Fig. 4F).

Even in all 6 cells, some adipocytes were not successfully transfected or reprogrammed, retaining the OP9 cells; however, anti-ER-TR7 response in all 6 cells was much more than that in MC cells. Collectively, these results suggested that adipocytes change into fibroblasts after transfection by the six master regulators.

## Discussion

In our study, we successfully reprogramed some mouse adipocytes into dermal fibroblast-like cells, although other adipocytes did not change. The rates of successful reprogramming were thought to depend on infection rates. We measured the transfection rates, using FACS, in terms of rates of GFP-positive cells, and the values were < 30‒40%. To achieve higher transfection rates, different factors of viral vectors were considered. We used retrovirus as a vector in this study. It was difficult to achieve higher transfection rates, since adipocytes, especially mature ones, generally have low proliferative capacities. To resolve this problem, changing the vector-containing virus into other viruses (such as lentivirus) may be useful in the future.

In various previous reprogramming studies, the number of master regulators for successful reprogramming was kept under three or four^5–8^. However, in our study, six master regulators were used. Among these six genes, some core genes might work effectively for reprogramming, while others may not. Identifying these effective core genes may yield better results. For identifying the core genes, different combination patterns of the six master regulators should be examined, although the roles of these genes in the differentiation of dermal cells still remain unknown. We have already examined some simple combination patterns of these genes, such as removal of one gene from the six and using only five genes; however, under low transfection rates, as mentioned above, comparison of the results with those of whole genes is difficult. Identifying candidate core genes, for achieving higher transfection rates, and assessing various combination patterns of the six genes may help in future studies.

Our study may provide one of the effective treatments for deep burns in future. In case of deep burn, skin grafting is often selected to achieve early burn wound closure. Skin grafting includes full-thickness and split-thickness skin grafting. Full-thickness skin grafting includes both the epidermis and dermis, and is recommended for functionally important areas in preference to split-thickness skin grafting in postburn contracture release^9^. However, in extensive TBSA deep burns, full-thickness skin grafting is difficult to perform due to lack of donor sites. Split-thickness skin grafting includes epidermis and partially dermis, and it is used frequently for deep burns. Similar to full-thickness skin grafting, split-thickness skin grafting is limited by the lack of donor sites, especially in extensive TBSA deep burns. To resolve this problem, mesh techniques (expansion of graft) are often used. However, meshed skin grafts have aesthetic problems and need longer healing time than do unmeshed skin grafts. Therefore, application of meshed skin grafts is limited to areas where aesthetic considerations are not so important. Use of a skin substitute is also a good option, with improved long-term and functional outcomes^10–14^. However, when skin substitutes are used for permanent skin cover, aesthetic issues need to be considered. Cost considerations also cannot be ignored, especially in extensive TBSA burns. Moreover, different types of skin substitutes have unique advantages and disadvantages, and there is no typical skin substitute for cases of deep burns. Extracorporeal shockwave therapy may be effective in revascularization and healing in patients with burn and trauma^15^. Keratinocyte cell spray has shown comparable aesthetic and functional outcomes to split-thickness skin grafting in a randomized trial^16^. However, no prior study has shown any therapy superior to skin grafting in healing burns. Our cells may be one of the effective adjunctive therapies for future burn care. In small, yet deep burns, transplantation of our cells may be enough for healing. In case of extensive TBSA deep burns, combination of transplantation of our cells and skin grafting may be effective. Rejection responses may be avoided using self (donor)-adipocytes that remain intact, despite the deep burns. Our cells may resolve the existing problems not only in the acute burn phase but also in the chronic burn phase. Even in deep burns, “recipient” adipocytes may be taken from donor sites; therefore, aesthetic problems, especially in facial burns, resulting in differences in skin color or thickness, can be avoided. Therefore, skin matched to donor sites can be reproduced using our reprogrammed cells.

To display the effectiveness of our cells, in vivo experiments and transplantation of our cells to animal burn models should be performed in the future.

In conclusion, we successfully reprogrammed dermal fibroblast-like cells from adipocytes by transfecting six master regulators (ELF4, FOXC2, FOXO1, IRF1, PRRX1, ZEB1) of mouse. Our cells might provide a new treatment strategy for both the acute and chronic phases of deep burn treatment in the future.

## Methods

All animal-related procedures were approved by the Laboratory Animal Care and Use Committee of Keio University (Assurance No. 17061) and conducted in accordance with the guidelines of the National Institutes of Health, USA.

The genetic modification protocol in this study was approved by the Keio University Safety Committee for Recombinant DNA experiments.

### Cell culture

Positive control cells were obtained from C57/BL6J male mouse tails, and cultured using general methods.

Bone marrow stromal cells (OP9 CRL-2749, American Type Culture Collection, 10801 University Boulevard Manassas VA, USA) were cultured according to the manufacturer’s instructions. Briefly, cells were maintained in minimum essential medium (MEM)-α (135-15175 FUJIFILM Wako Pure Chemical Corporation, Osaka, Japan) supplemented with 20% FBS (premium fetal bovine serum, 171012, Nichirei Biosciences Inc, Japan) and 1% penicillin G (10,000 U/ml)-streptomycin sulfate (10,000 mg/ml) (PS). The medium was renewed every 3 to 4 days. At the first passage, 3 × 10^6^ cells were seeded onto 6-well plates (9.6 cm^2^ per well) with 3 ml medium and cultured for 4 DIV.

### Differentiation of adipocytes

OP9 cells were plated at 5000 cells/cm^2^. When cells adhered to the plate, the propagation medium was replaced with insulin oleate medium: MEM-α with 0.2% FBS, 175 nM insulin, 900 μM oleate bound to albumin (in 5.5:1 molar ratio, prepared as described previously)^17,18^, 100 U/ml penicillin, and 100 μg/ml streptomycin.

### Construction of retroviruses

The pMXs retroviral vectors containing GFP, ELF4, FOXC2, FOXO1, IRF1, PRRX1, or ZEB1 were generated as follows. Plasmid DNA was sub-cloned into pDONR 201 vector, and subsequently cloned into pMXs-Gw, using the Gateway cloning system (Invitrogen, CA, USA) to generate pMXs-gene. The pMXs retroviral vectors were transfected into Plat-E cells using Fugene 6 (E2691, Roche, Basel, Switzerland) to generate retroviruses. Virus-containing supernatants were collected after 48 h^19^.

### Oil Red O staining

A stock solution of Oil Red O (0.5 g in 100 ml isopropanol) was prepared and passed through a 0.2-μm filter. Six milliliters of the stock solution was mixed with 4 ml of distilled water, left for 1 h at room temperature (25 °C), and filtered through a 0.2-μm filter prior to use. Cells were washed thrice with PBS, fixed with 10% formalin for 1 h at 4 °C, and stained with the Oil Red O working solution for 20 min at room temperature^20^.

### IncuCyte live-cell imaging

Fluorescence images were obtained every 3 h using an IncuCyte Live-Cell Imaging System (Essen BioScience, Ann Arbor, MI, USA).

### RNA extraction, cDNA synthesis, PCR, and real-time PCR

Total RNA was isolated using an miRNeasy Mini Kit (217004 Qiagen, Valencia, CA, USA) and converted to cDNA using the High-Capacity Reverse Transcription Kit (Applied Biosystems, CA, USA) according to the manufacturer’s instructions. Real-time PCR was performed using a QuantiFast SYBR Green PCR Kit (Qiagen) according to the manufacturer’s protocol. Primer sequences (5′ → 3′) are listed in Table 1. Expression levels were calculated using the 2^−ΔΔCt^ method and normalized to levels of the internal control glyceraldehyde-3-phosphate dehydrogenase (GAPDH).

**Table 1:** Primers for quantitative real-time PCR.

Gene	Sequence
**PPARγ2**	
Forward	TTTTCCGAAGAACCATCCGATT
Reverse	ATGGCATTGTGAGACATCCCC
**C/EBPα**	
Forward	GCGGGAACGCAACAACATC
Reverse	GTCACTGGTCAACTCCAGCAC
**aP2**	
Forward	ATCAGCGTAAATGGGGATTTGG
Reverse	GTCTGCGGTGATTTCATCGAA
**Leptin**	
Forward	GAGACCCCTGTGTCGGTTC
Reverse	CTGCGTGTGTGAAATGTCATTG
**Thy1**	
Forward	TGCTCTCAGTCTTGCAGGTG
Reverse	TGGATGGAGTTATCCTTGGTGTT
**VCAM1**	
Forward	AGTTGGGGATTCGGTTGTTCT
Reverse	CCCCTCATTCCTTACCACCC
**Vimentin**	
Forward	CGGCTGCGAGAGAAATTGC
Reverse	CCACTTTCCGTTCAAGGTCAAG
**Collagen1**	
Forward	GCTCCTCTTAGGGGCCACT
Reverse	CCACGTCTCACCATTGGGG
**Actin**	
Forward	CCCAGACATCAGGGAGTAATGG A
Reverse	TCTATCGGATACTTCAGCGTC
**GAPDH**	
Forward	AGGTCGGTGTGAACGGATTTG
Reverse	TGTAGACCATGTAGTTGAGGTCA

### Immunofluorescence

Cells were incubated with one of the following primary antibodies diluted in 0.1% BSA/PBS: anti-fibroblast marker (ER-TR7) (sc73355, Santa Cruz Biotechnology, TX, USA, 1:100), anti-CD90 (ab3105, Abcam, Cambridge, UK, 1:300), or anti-FABP4 (EPR3579) (ab92501, Abcam, 1:1000). After washing with PBS/0.1% Tween 20 (Wako Pure Chemical Industries, Japan), sections were incubated with Alexa Fluor 488-conjugated (Invitrogen, CA, USA, 1:500) or TSA Fluorescein System (NEL 702, PerkinElmer Life Sciences Inc, MA, USA) secondary antibodies for 1 h at room temperature. After counter-staining with Hoechst 33258 (94403, Sigma-Aldrich, MO, USA) to visualize nuclei, images were obtained with BZ9000 (Keyence, Japan). Stained cells were observed under a BioRevo BZ-9000 microscope (Keyence), and the number of fluorescence-positive cells was counted using the MicroCell Count software (Keyence).

### Statistical analysis

The results are presented as the means + SEM. Comparisons were performed using a two-tailed Student’s t-test. P < 0.05 was considered significant.

## Data Availability

The datasets generated during and/or analysed during the current study are available from the corresponding author on reasonable request.
